# The Spread of Dengue in an Endemic Urban Milieu–The Case of Delhi, India

**DOI:** 10.1371/journal.pone.0146539

**Published:** 2016-01-25

**Authors:** Olivier Telle, Alain Vaguet, N. K. Yadav, B. Lefebvre, Eric Daudé, Richard E. Paul, A. Cebeillac, B. N. Nagpal

**Affiliations:** 1 Centre National de la Recherche Scientifique, Unité de Recherche Associée 8204 Géographie-cités, Paris, France; 2 Institut Pasteur, Functional Genetics of Infectious Diseases Unit, Department of Genomes and Genetics, Paris, France; 3 Centre National de la Recherche Scientifique, Unité Mixte de la de Recherche 6266, IDEES, Rouen, France; 4 Centre de Sciences Humaines, Delhi, India; 5 National Institute of Malaria Research, Delhi, India; 6 Municipal Corporation of Delhi, Delhi, India; University Hospital San Giovanni Battista di Torino, ITALY

## Abstract

**Background:**

Dengue is a major international public health concern, one of the most important arthropod-borne diseases. More than 3.5 billion people are at risk of dengue infection and there are an estimated 390 million dengue infections annually. This prolific increase has been connected to societal changes such as population growth and increasing urbanization generating intense agglomeration leading to proliferation of synanthropic mosquito species. Quantifying the spatio-temporal epidemiology of dengue in large cities within the context of a Geographic Information System is a first step in the identification of socio-economic risk factors.

**Methodology/Principal Findings:**

This Project has been approved by the ethical committee of Institut Pasteur. Data has been anonymized and de-identified prior to geolocalisation and analysis. A GIS was developed for Delhi, enabling typological characterization of the urban environment. Dengue cases identified in the Delhi surveillance system from 2008 to 2010 were collated, localised and embedded within this GIS. The spatio-temporal distribution of dengue cases and extent of clustering were analyzed. Increasing distance from the forest in Delhi reduced the risk of occurrence of a dengue case. Proximity to a hospital did not increase risk of a notified dengue case. Overall, there was high heterogeneity in incidence rate within areas with the same socio-economical profiles and substantial inter-annual variability. Dengue affected the poorest areas with high density of humans, but rich areas were also found to be infected, potentially because of their central location with respect to the daily mobility network of Delhi. Dengue cases were highly clustered in space and there was a strong relationship between the time of introduction of the virus and subsequent cluster size. At a larger scale, earlier introduction predicted the total number of cases.

**Conclusions/Significance:**

DENV epidemiology within Delhi has a forest fire signature. The stochastic nature of this invasion process likely smothers any detectable socio-economic risk factors. However, the significant finding that the size of the dengue case cluster depends on the timing of its emergence emphasizes the need for early case detection and implementation of effective mosquito control. A better understanding of the role of population mobility in contributing to dengue risk could also help focus control on areas at particular risk of dengue virus importation.

## Introduction

Dengue is a tropical and sub-tropical disease whose etiological agent is a virus (the dengue virus, DENV), which is transmitted by mosquitoes. The major mosquito vector is *Aedes aegypti*, which has adapted to urban conditions; *Aedes albopictus* is a secondary vector, occurring in more rural environments and notably recently invaded Europe (www.vbornet.eu). Although the disease has been known for several centuries [1], rigorous study of DENV epidemiology only started after World War II in the Pacific Islands and South East Asia [2–4]. Today, an estimated 70–500 million people are infected by the virus every year in over a hundred countries across the world [5]. In South East Asia, the disease has been one of the major causes of hospitalisation among children since the 1990s [6]. Although the vector and the disease are currently concentrated in tropical and inter-tropical areas, the spread of competent mosquito vectors and increased population movement may lead to the virus becoming endemic in temperate areas [7]. Dengue has already been detected in Argentina [8] as well as in Europe (Portugal, Croatia, France), where autochthonous cases have already occurred [9–10]. In the absence of an effective vaccine [11], effective vector control by eliminating adult mosquitoes and removing potential egg laying sites is the only way of checking the spread of the virus once there has been an outbreak of the disease.

The majority of studies to date have focussed on the intra-urban spread of dengue under epidemic conditions [12–17]. Most of these studies have identified a dual dissemination of the infection: at the hyper-local level, dengue cases spread from an index case into its immediate environment (less than 1 sq. km.), whereas at the macro level, individual mobility leads to relocalisation of the virus towards more distant areas. However, while these studies have described the spread of dengue in detail and identified contributing factors, three main questions remain unanswered: Can a relationship between space (the concentration of cases in one area) and time (early introduction of the virus) be identified? Do there exist permanent clusters in an urban setting–i.e. clusters that are detected every year? Can we detect a relationship between socio-economical quality of the environment and dengue incidence? Here we address these three points in an area of hyperendemic dengue in Delhi over a three year period.

## Study Area

**Fig 1:**
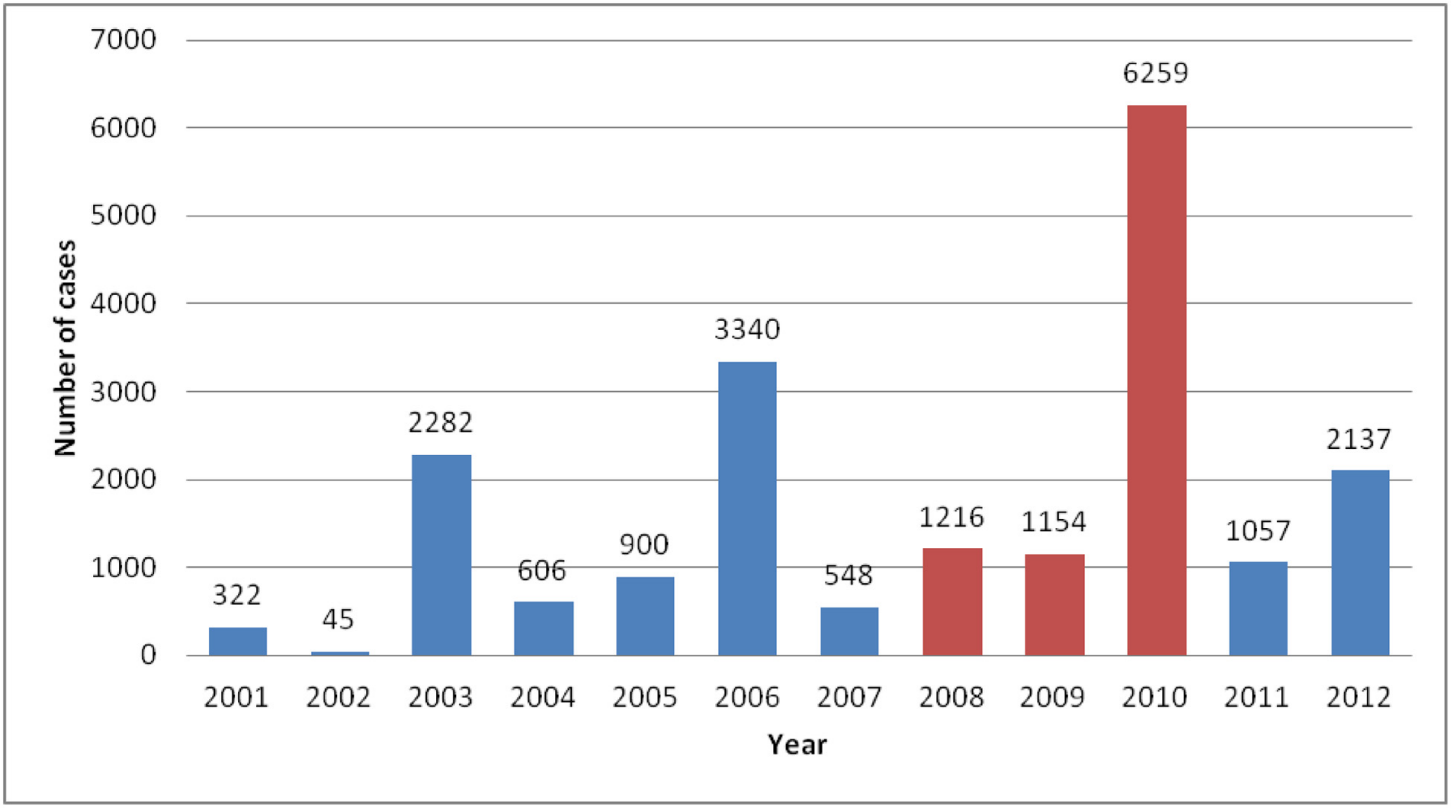
Number of dengue cases reported in Delhi between 2001 and 2010 (source: MCD). Studied years in this article are in red.

The situation of dengue in India is relatively unknown, although, according to estimations, the country hosts two-thirds of the population at risk globally and infections could be higher in the sub-continent than anywhere else [5]. This study analyses the spread of the dengue cases in Delhi, India. The Indian metropolis of 16.7 million inhabitants (Census 2011) has recorded dengue cases every year since 1996. Although Delhi experienced dengue several times in 1970s and 1980s, 1996 remains the year that witnessed the outbreak of an epidemic of unprecedented proportions with around 10,252 cases in Delhi with a 4.1% mortality rate [18–23]. This was also the year in which the current surveillance system was implemented. Since 1996, Delhi has become officially the most dengue-affected city in India even though this over representation of the metropolis in official dengue figures is likely linked to a better surveillance system. In addition, dengue in Delhi is hyper-endemic and not just endemo-epidemic (a temporary large increase in the number dengue cases), with all four viral serotypes periodically co-circulating since 2003 [24]. Between 2001 and 2011, three dengue epidemics were reported, with between 2,800 and 6,200 cases; in the years when no epidemic was declared, the incidence oscillated between 45 and 1,216 cases per year (Fig 1). The case fatality rate was 0.69% for this 10 year period (140 deaths for 20,289 cases).

In this article, we study the location of dengue cases reported in 2008, 2009 and 2010. We have chosen to base our study on these three years for two main reasons. First, as Delhi reported approximately the same number of cases during 2008 and 2009, the role of geographical factors can be more easily compared. Secondly, after having observed a circulation of serotypes 3 and 2 from 2004 to 2007, Delhi seemed to be the hub of an almost exclusive spread of serotype 1 in 2008 [25]. Although the population of Delhi was exposed to serotype 1 in 1997 and 2003, it did not persist within Delhi and the population thus remained largely naive to this serotype.

## Materials and Methods

### Dengue data

We based our research on a Geographic Information System (GIS) in order to localise dengue cases reported through the Delhi surveillance system in 2008, 2009 and 2010. One of the biases stemming from the use of hospital data may be due to surveillance system deficiencies: as is the case in most parts of India, dengue cases can not be correctly recorded in an urban milieu [26]. However, Delhi is probably the ideal Indian city in which to conduct such a study, because the dengue sentinel network consists of 33 public and three private hospitals (in 2010). This means that all public hospitals of more than 100 beds are included in the surveillance system. In comparison, at the same date, the state of Kerala had only 10, Tamil Nadu 13, West Bengal 10 and Bihar one sentinel hospital (http://www.nvbdcp.gov.in). Thus, the census in Delhi, with one sentinel hospital for 508,725 inhabitants in 2010, is much more reliable than other states, because there is, for instance, just one sentinel hospital for 3.3 million inhabitants in Kerala, and one for 104 million inhabitants in Bihar. Moreover, the other Indian metropolises have just one (Kolkata) or two sentinel hospitals (Chennai and Mumbai), even though surveillance system seems to have improved since 2010. Only confirmed cases are recorded in surveillance system, and only a small percentage of cases are screened.

After obtaining morbidity data, one of the difficulties we faced was to digitalize the addresses of patients affected by dengue. Dengue cases were confirmed for the presence of IgM antibodies against DENV by MAC ELISA using a kit prepared by the National Institute of Virology, Pune, India (as an integral part of the National Vector Borne Disease Control Programme), strictly following the manufacturer’s protocol [27]. Confirmed cases were then processed with ARCGIS 10.1. The data were classified on the basis of several parameters: gender, age group and the day the patient went in for consultation to one of the hospitals. Of the 1,312 dengue cases registered in 2008, 97% could be traced (i.e. 1,270 cases). In 2009 and 2010 this percentage remains stable with 1,129 cases found in 2009 (98%) and 5,998 in 2010 (96%) localized.

### Socio-economic characterization of Delhi

According to the last census (2011), the Indian capital has a population of 16.8 million inhabitants (National Capital Territory of Delhi). Rapid urban and demographic growth coupled with poor urban planning and decades of underinvestment in urban infrastructures (water, waste etc) have created striking social and environmental disparities across the metropolis [28–30]. This spatial fragmentation of Delhi is not easily captured by official data that are only available at aggregated levels such as wards (N = 289, average population = 58,000) or districts (N = 9, average population = 1.9 M.) from Census of India and local authorities (municipal corporations, Union State). In order to assess risk factors at the local level, we collected data on environmental parameters for each of the 1,280 colonies registered in 2008. Colonies are administrative spatial units that are used for property tax collection and public investment. Based on several variables assessing the level of infrastructure and access to urban services, property tax varies from one colony to another through a Unit Area Value system (GNCTD, 2010). Integrating data on land use, population density and property tax ranking for each colony in a GIS enables us to have an estimation of local socio-environmental heterogeneity.

Each colony is allotted a property tax score (out of 100) composed of 10 criteria (criteria noted out of 10): access to physical urban infrastructures (such as water, quality of road) and social infrastructures (such as presence of a hospital), the age of the colony, its type (approved, non-approved, recently approved, etc.); economic status of residents, access to roads, locative and rent values of the property, access to commercial centers and colony location in Delhi. Each criterion of the property tax system is decomposed into three levels: 10 if the rank is good, six for middle and three for very low. We then chose four variables of the property tax to compose a socio-economic map of Delhi regarding estimated factors of risk that are associated with classical diseases: level of infrastructure, economic status of resident, type of colony and total property tax score. Some other variables were also integrated: estimated population per unit and, in order to exclude industrial areas from residing colonies, % of the land use dedicated to industry. Population size was estimated through the use of official data (census 2011) and remote sensing (Spot 2011). We use a “unit” to gather all the information available. This technique enables the use of homogeneous units in term of size and is an efficient technique to gather information at different scales; we chose a 250m*250m unit. We then carried out a Principle Components Analysis on the six variables used to qualify the environment (typology) to create homogeneous groups of the 10,676 units that compose the urban area of Delhi (see S1 Fig).

### Risk factor analysis

The number of dengue cases occurring in a unit was analyzed by poisson regression using Genstat ver. 15 (VSN Ltd). A dispersion parameter was estimated and Wald statistics, which approximate to a χ^2^ distribution, were established. First we combined all three years and assessed the impact of typology, distance from the forest and distance to the nearest sentinel hospital, taking into account the population size in the unit as variables. Typology was considered as a category and distance to forest and sentinel hospitals as continuous variables. We then analyzed, by poisson regression, each year separately to assess the effect of proximity to an index case on the number of dengue cases; the index cases were thus removed from the analysis. We considered the first 50 cases appearing in Delhi as city-level index cases. Thus index cases included cases occurring within 3 weeks after the first case registered for each year. The above distance, typology and population size variables were used. Proximity to index cases was divided into the following distance groupings:] 0m -] 100m,] 100 -] 250m -] 250 -] 500m,] 500m -] 750m,] 750m -] 1000m,] 1000m -] 1500m and] 1500m. Finally we analysed by logistic regression, the effect of typology on the probability of a dengue index case, as defined above, being detected in a unit. We decided to observe the spatiality of the index cases to observe if during the 3 years index cases were more prone to emerge from a specific typology of areas.

### Spatial and spatio-temporal analyses of cases

Spatial analyses were performed with ArcGIS 10.1^™^. To reveal the spatial structure of DENV transmission, we analysed clustering through the K-Ripley index. The global clustering examined the spatial pattern of dengue cases at different steps (min = 50 meter radius, max = 500 meters with a 50 meter step). To compare observed results of N dengue cases (in year x) with a random distribution, we carried out 999 Monte Carlo simulations on all dengue data (2008, 2009 and 2010) to obtain respective minimum and maximum confidence intervals of N random pattern. If the K-Ripley score was above the upper confidence interval, dengue cases are clustered, whereas cases are over-dispersed with a score below the lower confidence interval.

Local detection of dengue intensity was performed through kernel density estimation (KDE). This tool enables spatial detection of spatial intensity of dengue cases. KDE is computed for each unit cell (here each unit is 250m by 250m) [31]. We used a search radius such that only cases that fall within this radius contribute to the estimation of Kernel density for each unit. Although in a classical setting the bandwidth is defined through the K-function maximum score, we used here a 750m search radius; a smaller threshold would make the map very difficult to read due to the size of the territory (2000 km^2^).

While KDE identifies a concentration of cases, it does not link the cases in time: two individuals may be close in space, but distant in time, suggesting that the two infections are unlikely to be linked. Spatio-temporal analyses enable linking of the cases in time and space: if two cases occur within pre-determined limits, there is a high probability that one of them was an infection either arising from the same pool of infected mosquitoes (if infection occurred within 15 days maximum) or due to a secondary infection (naive mosquito that became infected from a primary human infection). We performed the spatio-temporal analyses with R^™^ (Rgdal and SP package) to cases according to spatial and temporal windows. To detect spatio-temporal clusters, we used a spatial limit of 300 meters and a temporal window of 21 days, which corresponds to the DENV lifecycle [4]. Trials using distances from the selected dengue case varying from 100m and 500m either generated too few cases (100m) or very few clusters of very large size. 300m gave the best resolution and was deemed reasonable given the average distance of mosquito flight.

Analysis of cluster size and time (day) during the epidemic was carried out by first normalizing the day with respect to the day of the first detected case and then fitting a loglinear regression of number of cases against day since the start of the epidemic taking into account the year. We also fitted a non-linear Gaussian curve to the number of cases against day of the year to estimate the epidemic peak. We then analysed the relationship between number of cases and day, separating the time periods prior to and post-peak of the epidemic. As above, analyses were performed using Genstat ver. 15 (VSN Ltd) and a dispersion parameter was estimated and Wald statistics, which approximate to a χ^2^ distribution, were established.

## Results

### General Dengue Data

Over the three years, more males were affected (2.5 male for 1 female reported), but since Delhi has a much greater male than female population (870 females for 1000 males), the ratio falls to 1.5 males/1 female. The most affected age group was 26–59 years old (35.5% of cases), followed by those in the 15–25 year category. A distinct period of dengue activity occurred from August to December, during and especially after the monsoon in 2008, 2009 and 2010 (see S2 Fig). The first case in 2008 was registered on the 19^th^ July, 33 days after the first substantial rain that affected Delhi on the 6th June. In 2009, even though the first case was registered at the same time as in 2008 (7th July), cases only started to spread in September, likely because of the late and relatively weak monsoon in July. In 2010, dengue diffusion started much earlier than in 2008 and 2009 (first cases 6th of June), even though rainfall started later compared to 2008.

### Environmental characterization of Delhi

**Fig 2:**
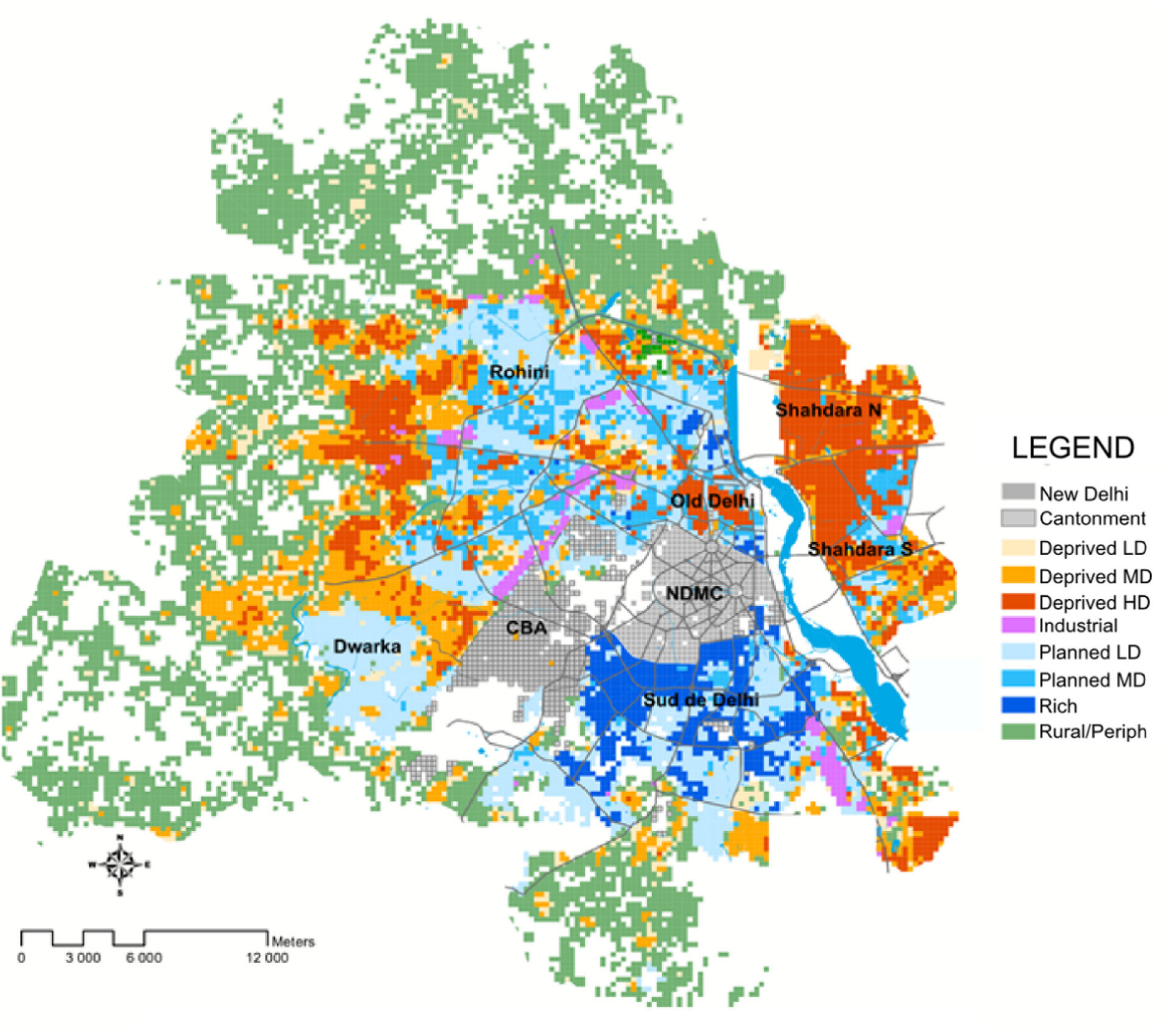
Map representing environmental typology of Delhi

The environmental characterization revealed that 42% of units were deprived areas, characterized by poor access to urban infrastructure and a high population density. It is of note that some parts of the city, the military camp (named CBA or Delhi Cantonment) and New Delhi (NDMC), do not use the same property tax system. We decided to include them in the analysis as independent units even though these two areas concentrate only 3% of the population. New Delhi can be considered as a well planned area and quite similar to "rich" units. See Fig 2 for a representation of environmental typology of Delhi.

### Impact of urban typology on dengue risk

In a first analysis we examined the association of the number of dengue cases in the unit at any time over the three years with the typology, the distance with respect to the nearest forest as well as to sentinel hospitals (continuous predictor), all the while controlling for the population size in the unit (as a covariate). Of the 10,676 units, 3850 had dengue cases during at least one year, 522 had dengue cases in 2 years and 93 had cases every year. Both distance variables were initially treated as categories. The typology type "periphery" had the lowest dengue incidence rate (Table 1) and was treated as the reference level.

**Table 1:** Population, total number of dengue cases and mean incidence rate (per 100,000) in 2008, 2009 and 2010 per typology of area.

Typology	Number of inhabitants	Dengue cases 2008	Incidence rate (mean per units)	Dengue cases 2009	Incidence rate	Dengue cases 2010	Incidence rate
Deprived Low Density	1 862 995	138	5.71	159	5.50	970	40.1
Deprived High Density	1 632 073	88	7.36	67	5.93	357	32.9
Deprived Medium Density	7 762 975	617	9.56	437	6.25	2554	43.1
Delhi Cantonment	113 079	28	8.06	16	13.13	147	59.4
Rich	753 774	101	13	138	18	546	72
Indus	218 589	14	5.72	4	3.91	33	12.2
Periph	474 287	24	4.10	39	5.00	179	24.0
Planned	2 952 736	260	9	264	9	1 213	41
Total Delhi	15 770 507	1 270	8.75	1129	8.89	5998	46.5

Delhi Cantonment and Indus units had significantly lower numbers of dengue cases than peripherical units over the 3 year period. Dengue cases were higher in almost all the other typologies, increased with population size (χ^2^_1_ = 379.3, P<0.001) and decreased with distance from forest (χ^2^_1_ = 323.6, P<0.001) (Table 2) and distance to sentinel hospitals (χ^2^_1_ = 23.8, P<0.001). Deprived low density units had lower odds ratios than Deprived Medium and High Density units and Rich units (Table 2).

**Table 2:** Association of environmental and socio-economic factors with number of dengue cases per unit area over 3 years. ORa–adjusted Odds Ratio for unit increase in dengue cases. CL—Confidence Limit. Population is taken as a predictive value.

	ORa	Lower 95% CL	Upper 95% CL	p
**Continuous predictor**				
Population	**1.00009**	**1.00008**	**1,00010**	**0,00**
Distance to Forest	**0.99990**	**0.99989**	**0,99992**	**0,00**
Distance to Sentinel Hospital	**0.99998**	**0.99997**	**0,99999**	**0,00**
**Typology**				
Rich	**2.96**	**2.50**	**3,51**	**0,00**
Planned	**2.81**	**2.39**	**3,30**	**0,00**
Deprived LD	**2.47**	**2.10**	**2,91**	**0,00**
Deprived MD	**6.49**	**5.54**	**7,59**	**0,00**
Deprived HD	**7.27**	**6.04**	**8,75**	**0,00**
Indus	1.15	0.81	1,64	0,44
Cantonment	**2.03**	**1.64**	**2,52**	**0,00**
Periph (rural)	**REF**			

We then analyzed each year independently and assessed the extent to which distance from an index case influenced the number of dengue cases per unit that year. Distance from the forest was fitted as a continuous variable and typology periphery was used as the reference level. The odds ratio of a unit increase in the number of dengue cases occurring in proximity to an index case decreased rapidly, from a distance as little as 100m in 2010 and 2008, and 250m in 2009 (Table 3). The incidence of dengue decreased with increasing distance from the forest all three years (2010 χ^2^_1_ = 66.2, P<0.001; 2009 χ^2^_1_ = 26.1; 2008 χ^2^_1_ = 23.1, P<0.001). Dengue incidence increased with population size in all three years (2008 χ^2^_1_ = 194.5, P<0.001; 2009 χ^2^_1_ = 185.3, P = 0.001; 2010 χ^2^_1_ = 70.6, P<0.001). With respect to typology, Deprived Medium and High density units had higher incidence of dengue all 3 years.

**Table 3:** Association of environmental, socio-economic factors and proximity to dengue index cases with number of dengue cases by year. Shown are adjusted Odds ratios (ORa) for continuous predictors and categorical variables in the final minimal adequate multivariate poisson regression model. In red: significant p-values.

Year	2010				2009				2008			
	Ora	Lower	Upper	p	Ora	Lower	Upper	P	Ora	Lower	Upper	p
Continuous Predictors					Continuous Predictors				Continuous Predictors			
Population	**1.0001**	**1.0000**	**1.0001**	**0.0000**	**1.0000**	**1.0001**	**1.0002**	**0.0000**	**1.0002**	**1.0002**	**1.0002**	**0.0000**
Distance to forests	**0.9999**	**0.9999**	**0.9999**	**0.0000**	**1.0000**	**0.9999**	**0.9969**	**0.0018**	**0.9999**	**0.9999**	**0.9999**	**0.0000**
Distance to sentinel hospitals	**0.9999**	**0.9999**	**0.9999**	**0.0000**	1.0000	1.0000	1.0000	0.1439	1.0000	1.0000	1.0000	0.2255
Unit typology					Unit typology				Unit tpology			
Rich	**2.87**	**2.31**	**3.57**	**0.00**	**1.61**	**1.17**	**2.20**	**0.00**	**1.89**	**1.32**	**2.72**	**0.00**
Planned	**3.02**	**2.45**	**3.71**	**0.00**	1.28	1.16	1.71	0.09	**1.82**	**1.30**	**2.55**	**0.00**
Deprived LD	**3.08**	**2.50**	**3.80**	**0.00**	1.17	1.16	1.57	0.29	**1.43**	**1.00**	**2.03**	**0.05**
Deprived MD	**8.04**	**6.57**	**9.85**	**0.00**	**1.66**	**1.16**	**2.21**	**0.00**	**2.50**	**1.79**	**3.49**	**0.00**
Deprived HD	**10.03**	**7.95**	**12.67**	**0.00**	1.50	1.24	2.29	0.06	**2.19**	**1.41**	**3.40**	**0.00**
Indus	0.95	0.59	1.55	0.85	1.13	1.35	2.04	0.67	0.53	0.19	1.49	0.23
Cantonment	**2.37**	**1.81**	**3.09**	**0.00**	1.34	1.22	1.99	0.15	1.00	0.56	1.79	1.00
Periph (rural)	REF				REF				REF			
Distance to Index cases					Distance to Index cases				Distance to index cases			
]0m -] 100m	**3.14**	**2.75**	**3.58**	**0.00**	**3.57**	**1.16**	**4.78**	**0.00**	**4.55**	**3.40**	**6.10**	**0.00**
]100m -] 250m	**2.23**	**1.95**	**2.56**	**0.00**	**4.49**	**1.12**	**5.63**	**0.00**	**3.52**	**2.68**	**4.62**	**0.00**
]250m -] 500m	**1.39**	**1.23**	**1.56**	**0.00**	**1.69**	**1.13**	**2.16**	**0.00**	**2.48**	**1.97**	**3.11**	**0.00**
]500m -] 750m	**1.50**	**1.35**	**1.66**	**0.00**	**1.60**	**1.12**	**2.00**	**0.00**	**1.94**	**1.55**	**2.43**	**0.00**
]750m -] 1000m	**1.27**	**1.14**	**1.41**	**0.00**	**1.58**	**1.12**	**1.96**	**0.00**	**2.15**	**1.75**	**2.64**	**0.00**
]1000m -] 1500m	**1.17**	**1.08**	**1.27**	**0.00**	**1.45**	**1.09**	**1.71**	**0.00**	**1.05**	**0.80**	**1.36**	**0.73**
]1500m and more	REF				REF				REF			

Finally, the Table 4 shows that rich units are the more prone to detect a index cases compared with others units. In Delhi and as in almost all the urban areas worldwide, central areas remain mainly rich areas. This could explain that index cases are registered early in the epidemic season in these central areas due to their topologic situation at city scale, virus being spread quickly in these space at the beginning of epidemic.

**Table 4:** Association between unit typology and probability for a unit to detect an index cases during the three years, logisitic regression.

Unit Typology	OR	Lower	Upper	P
Deprived LD	0.72	0.41	1.26	0.25
Deprived MD	**2.45**	**1.50**	**3.99**	**0.00**
Rich	**3.61**	**2.17**	**5.98**	**0.00**
Delhi Cantonment	1.63	0.81	3.27	0.17
Planned	**1.84**	**1.12**	**3.02**	**0.02**
Deprived HD	**2.33**	**1.07**	**5.08**	**0.03**
Indus	0.68	0.16	2.94	0.60
Periph	REF			

### Overall clustering

**Fig 3:**
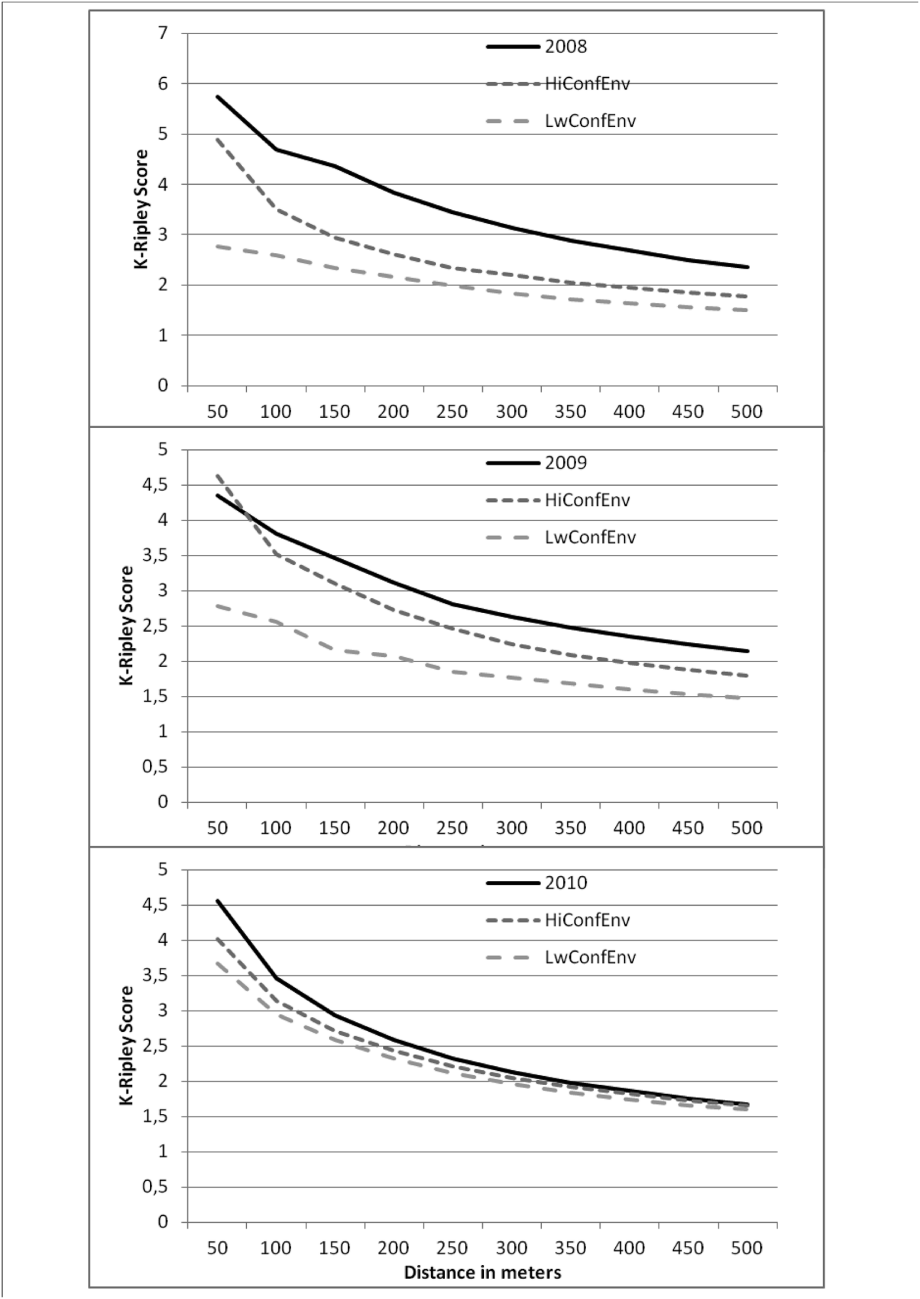
K-Ripley score for dengue cases and 99.9% upper CI and lower CI for number of cases expected under a random distribution.

To facilitate visual interpretation of the Ripley’s K-function, we plotted the difference between observed and expected score over the expected score. The K-Ripley analysis revealed that dengue cases were more clustered than expected (i.e. randomly distributed according to population or not). Dengue cases were highly clustered in space as the score was higher than the upper confidence limit and higher than the confidence interval (Fig 3). We observed a cluster peak at a radius of 50 meters in 2008 and 2010. However, in 2009, the peak observed at 50m cannot be excluded to be different from random, but other distances from 100m show higher than expected clustering. The results globally suggest that there is significant local diffusion of dengue virus.

### Kernel Density Estimation

**Fig 4:**
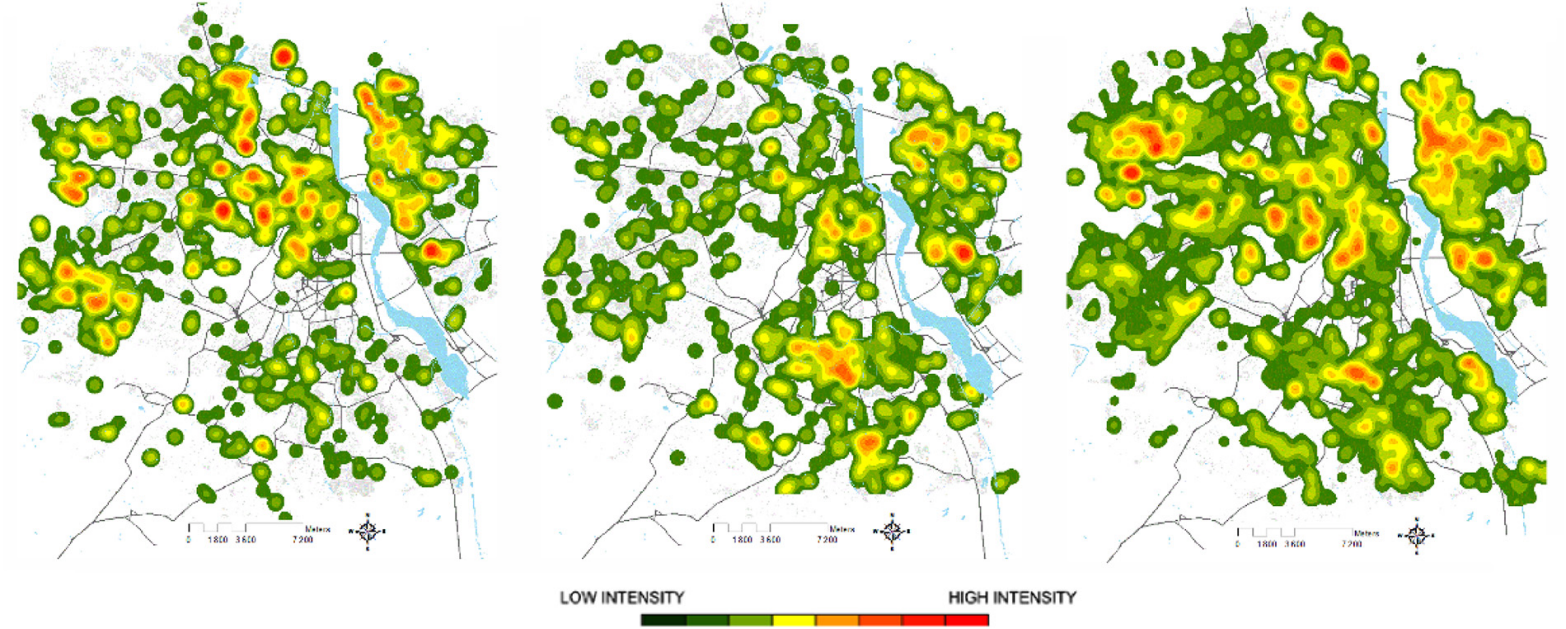
Kernel Density Estimation (KDE) of individuals registered in the sentinel hospitals of Delhi.

The KDE map (Fig 4) reveals that in 2008, the highest density of cases (last three quantiles that appear as red in the map) are located in West, Central and East Delhi. When we compare this map with the typology one (Fig 2), we can see that high density of dengue cases are mainly located in Deprived typology units. No important (i.e. in highest three quantiles) cluster is located in New and South Delhi. Although the 2009 KDE shows that Central and East Delhi are, as in 2008, affected, South Delhi, mainly composed of Rich and Planned units, have important densities of infected individuals in 2009. Globally, the KDE reveals a different geographical distribution of the disease in 2008 and 2009 despite a similar number of dengue cases in both years (1,253 and 1,129 cases). Thus, even though the system appears globally stable (same numbers of individuals infected), an instability is revealed at the meso-scale. During the weak epidemic years (i.e. in 2008 and 2009) KDE shows that individuals affected by dengue are located almost everywhere on the territory of Delhi, in concordance with the very marginal differences in risk associated with typology in the multivariate logistic regression (Table 3). However, during the 2010 epidemic, differences among categories tend to be intensified, especially for deprived areas, which are particularly more affected in 2010. The video of weekly KDE (for 2008, 2009 and 2010 see S1 File) shows an initial identifiable model of spread: after the registration of a first infected individual, an index case, other infected individuals were registered two to three weeks later in the majority of clusters identified in 2008. This local spread model corroborates the K-Ripley analysis that showed a strong clusterisation of cases in space. The video also suggests an important relationship between time, space and the number of infected individuals; the most important clusters were the ones emerging at the beginning of the outbreak.

#### Spatio-temporal clustering of cases

The spatio-temporal clustering model identified 195 clusters in 2008 comprised of 745 cases (58% of all cases) and 190 clusters in 2009 comprised of 669 cases (59% of cases) (Table 5). In all years, large clusters were associated with lower mean distance between cases, indicating higher case density. The duration of large clusters was also greater as might be expected. During 2010, an important decrease in the number of isolated cases was observed; only 17% of cases were isolated. See S3 Fig for localisation of these clusters.

**Table 5:** Spatio-temporal characterization of dengue case clusters.

**Number of cases in cluster in 2008**	**Number of cases in these categories of clusters**	**Mean distance between cases in metres**	**Min Distance in metres**	**Max Distance in metres**	**SD**	**Mean Duration in days**	**Min Duration in days**	**Max Duration In days**	**SD**
**Isolated individual**	549	450.3	24.3	4102.2	396.2	-	-	-	-
**2**	200	118.2	0.0	296.9	64.6	8.2	0.0	21.0	6.0
**3–4**	178	107.7	14.7	258.5	48.0	18.1	3.0	41.0	10.4
**5–6**	117	93.1	29.5	166.9	36.5	27.1	12.0	44.0	10.4
**7–9**	66	107.9	46.8	137.7	28.9	33.8	21.0	49.0	8.8
**10–12**	78	105.9	70.5	141.2	26.3	56.3	32.0	82.0	18.4
**14–17**	79	57.8	35.2	90.0	20.2	51.6	26.0	80.0	22.6
**Total cases**	1313	350.8	4.3	3351.2	326.1	5.6	0.0	71.0	10.1
**Number of cases in cluster in 2009**	**Number of cases in these categories of clusters**	**Mean distance between cases in metres**	**Min Distance in metres**	**Max Distance in metres**	**SD**	**Mean Duration in days**	**Min Duration in days**	**Max Duration In days**	**SD**
**Isolated individual**	457	472.8	23.5	3216.7	346.6	-	-	-	-
**2**	178	134.0	7.7	299.7	84.0	9.0	0.0	21.0	6.6
**3**	120	108.4	5.8	251.6	63.6	16.8	3.0	33.0	7.0
**4–5**	165	117.1	37.3	243.6	43.9	20.7	3.0	39.0	8.8
**6–7**	83	112.9	31.1	211.3	49.1	27.4	17.0	41.0	7.7
**8–11**	55	78.9	50.3	106.3	22.0	37.8	21.0	69.0	18.0
**12–15**	27	129.0	127.2	130.8	2.5	43.5	37.0	50.0	9.2
**20–21**	41	77.2	76.9	77.5	0.4	56.0	45.0	67.0	15.6
**Total cases**	**1126**	**369.6**	**5.8**	**3216.7**	**334.5**	**4.7**	**0.0**	**69.0**	**9.7**
**Number of cases in cluster in 2010**	**Number of cases in these categories of clusters**	**Mean distance between cases in metres**	**Min Distance in metres**	**Max Distance in metres**	**SD**	**Mean Duration in days**	**Min Duration in days**	**Max Duration in days**	**SD**
**Isolated individual**	1004	259.8	0.0	4001.6	249,5	-	-	-	-
**2–5**	1188	109.7	0.0	295.4	69,1	13.8	0.0	46.0	10.0
**6–14**	967	83.1	18.0	210.7	32	40.8	11.0	75.0	15.2
**15–27**	730	72.8	24.3	113.9	19,7	64.2	37.0	94.0	14.4
**29–48**	584	67.7	44.4	93.7	16,2	78.0	6.0	101.0	21.4
**57–76**	525	59.5	44.4	78.8	10,7	80.4	71.0	94.0	7.9
**91–130**	556	58.9	43.8	70.0	12,1	99.0	82.0	115.0	12.4
**206–216**	422	58.6	50.2	67.1	12	105.5	99.0	112.0	9.2
**Total cases**	**5976**	**199.0**	**0.0**	**4001.6**	**214,5**	**9.6**	**0.0**	**115.0**	**18.9**

There was a negative relationship between the size of the cluster and time since the first case of dengue was detected (χ^2^_1_ = 422.5, P<0.001; Odds Ratio for dengue case by day change = 0.982, 95% Confidence Intervals (CI) 0.981–0.984). Fitting a gaussian curve to the annual data yielded a significant relationship between day and number of cases (for each year separately) and generated an estimated day of epidemic peak (see S1 File): Day of the year 251 in 2008 (P<0.001, % variation explained = 41.6%); Day of the year 299 in 2009 (P<0.001, % variation explained = 55.4%); Day of the year 229 in 2010 (P<0.001, % variation explained = 53.6%). We then analysed the relationship between the cluster size and time dividing the data into pre- and post-epidemic peak. Loglinear regression of the relationship between the number of cases in the cluster and day prior to the epidemic peak revealed no significant relationship (χ^2^_1_ = 0.61, P = 0.33; OR for dengue case by day change = 1.00, 95%CI 0.995–1.01). However, analysis from the peak of the epidemic revealed a very strong negative relationship between day (post-peak) and number of cases within the cluster (χ^2^_1_ = 294.9, P<0.001; OR for dengue case by day change = 0.979, 95%CI 0.977–0.982).

Interestingly, the peak estimated by fitting a gaussian curve to the number of cases per cluster per day was earlier than when fitting by total cases per day: Day of the year 236 in 2008; Day of the year 250 in 2009; Day of the year 210 in 2010. Re-analysis of the cluster size-time association pre/post-peak using the estimated peak from data plotted per cluster per day did not significantly alter the associations (or lack of) found above.

## Discussion

In this study we sought to address three questions pertinent to dengue epidemiology in a hyperendemic urban setting as Delhi. Specifically, we examined whether there is a relationship between socio-economical quality of the environment and dengue incidence, whether there exists a relationship between space and time, whether there exist permanent clusters.

We found that increasing distance from the forest areas in Delhi reduced the number of dengue cases (Table 2); this may be linked to the relative shelter provided by the vegetation to mosquitoes. There was an inverse association with dengue incidence and proximity to the sentinel hospitals for all 3 years (table 2); this suggests that our study is not biased regarding spatial access to sentinel hospitals. Our analysis revealed that deprived areas with medium and high densities had the highest incidence of dengue, but also that rich units were more affected than deprived areas with low densities, even though the density of inhabitants is comparable between these two categories. This is counter intuitive since preliminary entomological studies revealed that houses in rich areas had fewer *Aedes aegypti* larvae [32]. Despite this important link between socio-economical characterization and the *Aedes* index, the analysis of dengue in Delhi reveals that risk of dengue cannot be predicted only on the basis of socio-economical factors. This may be explained by the fundamental importance of geographical centrality within the city, with respect to diurnal population mobility, rather than socio-economical status alone. An increased level of importation of infected individuals into a central area could lead to a local epidemic despite a lower *Aedes* density. As shown in Table 4, rich areas of the city has the higher risk to detect emerging index cases, followed by poor areas with high density.

The second result revealed that the majority of dengue cases were clustered in space (see K-Ripley analysis). This is in agreement with previous studies carried out in urban areas [12–17]. This means that even if infections can occur during daily mobility (at school, workplace, etc.), the majority of infections are contracted in the residing colony. One of the most original results of this study was to reveal a strong relationship between the introduction of the virus in one area and the subsequent cluster size; the earlier the day of (detectable) introduction, the bigger was the cluster. This association of cluster size and time, however, only applied for the period from the epidemic peak onwards and not pre-peak. Thus, this association most probably reflects the seasonal increase and decline in conditions favorable for dengue transmission, whether as a result of local herd immunity or climatic features impacting upon mosquito longevity and abundance. One notable feature was that the epidemic peak was earlier when cases were plotted per cluster and not simply summed up by day. One explanation is that the clusters then disperse cases but which are no longer exposed to conditions favorable for onward transmission and hence unable to generate further substantial clusters. Some studies have described this spread model as a local forest fire spread [33], grouping dengue cases together around the index case in Florida, Puerto Rico [34], Cairns [17] Hanoi [35], Bangkok [36] or in Guyana [12]. A forest fire signature can be defined as a local cluster of cases that then “burns” out but prior to this seeds adjacent areas, but at a distance too far for mosquito dispersal. Thus, as has been shown previously, human mobility clearly contributes to the dispersal of the virus [37–39], but the extent to which clusters of cases then develop will be dependent upon the climatic conditions.

The KDE estimation and the spatio-temporal analyses revealed a changing geography of dengue epidemics over the three years with no evidence of permanent clusters at a very local scale. Whilst 36% of the spatial units had dengue cases in one of the three years, only 5% had cases in two years and less than 1% had cases every year. The relatively low number of detected cases in 2008 and 2009 may have limited the power to detect permanent clusters. The putative absence of any permanent clusters is, however, an important issue for control of dengue, since mosquito/dengue control can not be planned on the basis of previous year’s geographical distribution of dengue. Successful local DENV invasion will be considerably affected by the stochastic probability of survival in and transmission from infected mosquitoes. One major factor that may be of particular importance at the local level is the development of monotypic herd immunity that can lead to a low local force of infection [40]. Moreover, there is increasing evidence that short-term heterotypic immunity can last for over a year, which would contribute to a low density of susceptible individuals at a very local level despite the invasion of a novel serotype [36, 41, 42].

The variation in the first areas subjected to dengue likely largely determines the subsequent meso-scale geographical distribution of dengue incidence. The first 2008 cases emerged in West, North East and Central Delhi while in 2009, the first cases occurred in South Delhi as well as in some part of East Delhi at the beginning of the dengue season. These areas subsequently accumulated the highest incidence over the season. This is important information to better control the spread of dengue. Focusing on targeted intervention of early clusters (that will occur prior to the epidemic peak) will likely impact significantly on the spread of dengue. Thus, development of a surveillance system that incorporates geolocalisation to identify clusters would be, in principle, beneficial.

In conclusion, we noticed in Delhi, India, that DENV epidemiology has, as elsewhere, a forest fire signature. Successful local spread will however depend on many factors, including the density of susceptible individuals, time of the local importation and successful development of the virus within the mosquito. The stochastic nature of this invasion process at a city scale likely smothers any classical socio-economic factors that usually lead the virus in urban areas. This will be further exacerbated by the continual importation of virus from outside of the city, and the constant relocalisation of the virus in the city. The significant finding that the extent of the dengue case clustering peaks prior to the epidemic peak does, however, suggest that incorporating a geolocalisation capacity into the surveillance system could yield a potentially more effective strategy at limiting the spread of dengue. The geographical distribution of dengue within the city also underlines the necessity to estimate mobility patterns at a city scale to better map the areas most visited and where deployment of mosquito intervention might be most usefully deployed. One continuing major issue, however, is the need to find an intervention strategy that is effective. Fumigation has proved ineffective [43] but novel methods based on deployment of novel formulations of long-lasting residual insecticides offer some hope.

### Limitation

One of the major limitations of our study is the dependence on the Delhi surveillance system to detect dengue cases. Although better than the rest of India, clinical case reporting will be subject to bias and to some extent affected by individual socio-economic status. Moreover and potentially a more significant problem is the fact that the majority of infections are sub-clinical and thus the clinical cases represent only a small fraction of the circulating viral infections. Prospective studies aimed at detecting the incidence of sub-clinical infections, their relative occurrence with respect to clinical infections and factors affecting this relative occurrence could help lead to methods to extrapolate from clinical cases to total DENV infections.

## Supporting Information

S1 Fig Unit-wise (250m*250m) environmental characterization of Delhi * Score on 10. **Score on 100. *** Source: Eicher Map. In italic: source, Property Tax MCD.
(TIF)

S2 Fig Number of dengue cases registered at hospital, rainfall (in mm) and mean temperature per day in 2008, 2009 and 2010 (TIF)

S3 Fig Spatio temporal clusters detected in Delhi in 2008, 2009 and 2010: size and emerging day for each cluster. (DOCX)

S1 File Figures showing the Gaussian fit of the number of cases per day per year (A-C) and the number of cases per cluster per day per year (D-F). (DOC)

S2 File Data used for this analyze. (XLSX)

S1 Zip Files Compressed/ZIP File Archive: Temporal Kernel video for 2008, 2009 and 2010 (every 2 weeks). (RAR)
