# Correction: The Spread of Dengue in an Endemic Urban Milieu-The Case of Delhi, India

**DOI:** 10.1371/journal.pone.0152847

**Published:** 2016-03-29

**Authors:** The PLOS ONE Staff

The authors are listed out of order. The publisher apologizes for the error. Please view the correct author order, affiliations, and citation here:

Olivier Telle^1,2,4^, Alain Vaguet^3^, N.K Yadav^6^, B. Lefebvre^3^, A. Cebeillac^3,4^, B.N. Nagpal^5^, Eric Daudé^3,4^, Richard E. Paul^1,2^

1 Centre National de la Recherche Scientifique, Unité de Recherche Associée 8204 Géographie-cités, Paris, France, 2 Institut Pasteur, Functional Genetics of Infectious Diseases Unit, Department of Genomes and Genetics, Paris, France, 3 Centre National de la Recherche Scientifique, Unité Mixte de la de Recherche 6266, IDEES, Rouen, France, 4 Centre de Sciences Humaines, Delhi, India, 5 National Institute of Malaria Research, Delhi, India, 6 Municipal Corporation of Delhi, Delhi, India

Telle O, Vaguet A, Yadav NK, Lefebvre B, Cebeillac A, Nagpal BN, et al. (2016) The Spread of Dengue in an Endemic Urban Milieu–The Case of Delhi, India. PLoS ONE 11(1): e0146539. doi:10.1371/journal.pone.0146539

In the Author Contributions section, Richard E. Paul (RP) should be listed as one of the persons who contributed reagents/materials/analysis tools.
