# Correction: Correction: The Spread of Dengue in an Endemic Urban Milieu-The Case of Delhi, India

**DOI:** 10.1371/journal.pone.0158931

**Published:** 2016-07-05

**Authors:** The PLOS ONE Staff

## Notice of Republication

This article was republished on June 10, 2016, to correct a textual error in the body of the correction article. Alain Vaguet was spelled incorrectly in the correction text, appearing as: Alain Vague. The correction article meant to specify only the order of authors. The publisher apologizes for misspelling the author name. Please download this article again to view the correct version. The originally published, uncorrected article and the republished, corrected article are provided here for reference.

## Supporting Information

S1 File Originally published, uncorrected article. (PDF)

S2 File Republished, corrected article. (PDF)
