# Supplementary figures and images for: The Spread of Dengue in an Endemic Urban Milieu–The Case of Delhi, India
Source: PLoS One. 2016 Jan 25;11(1):e0146539. doi: 10.1371/journal.pone.0146539 (PMC4726601; doi:10.1371/journal.pone.0146539)

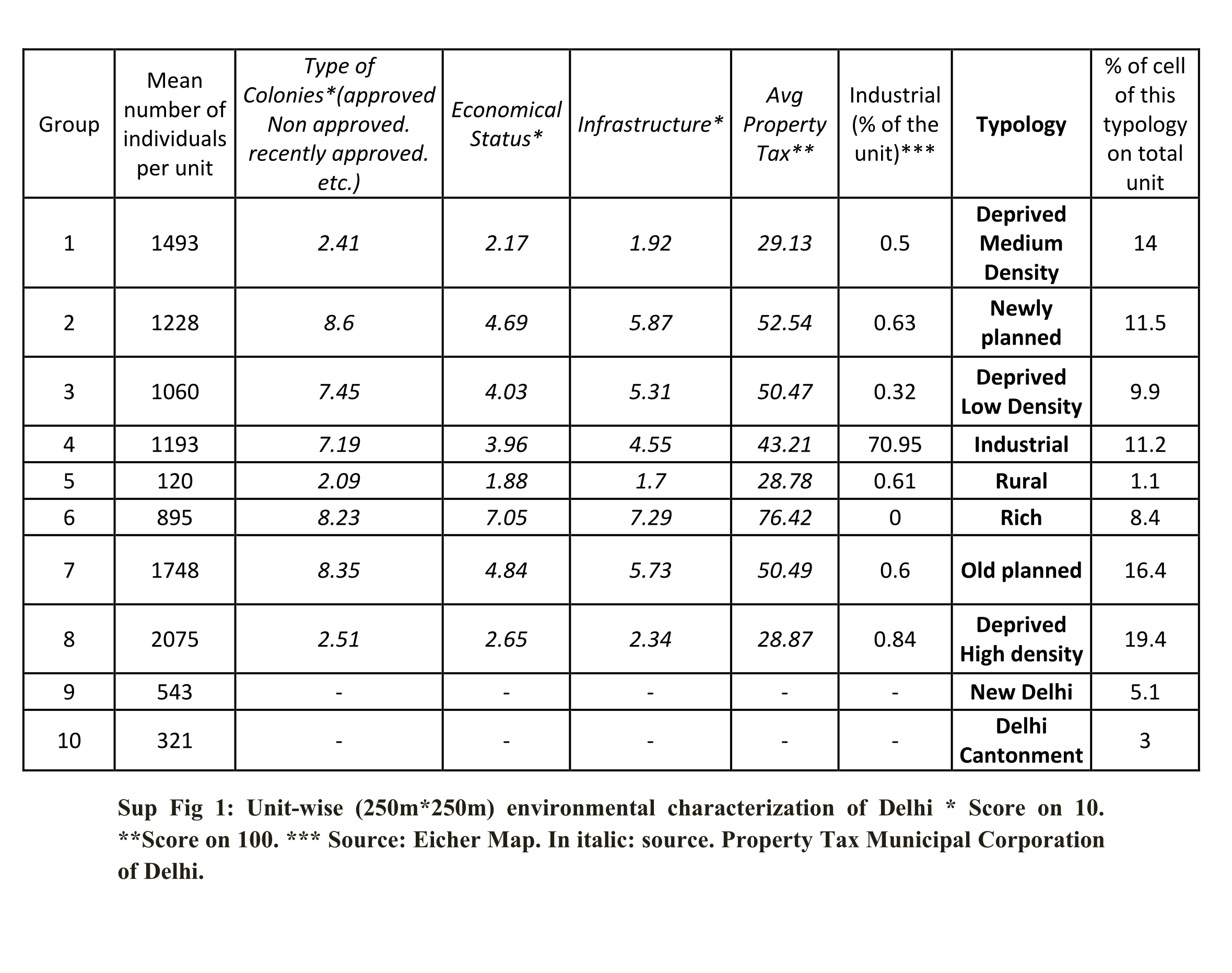

Supplement: S1 Fig — Unit-wise (250m*250m) environmental characterization of Delhi * Score on 10. **Score on 100. *** Source: Eicher Map. In italic: source, Property Tax MCD. (TIF) [file pone.0146539.s001.tif]

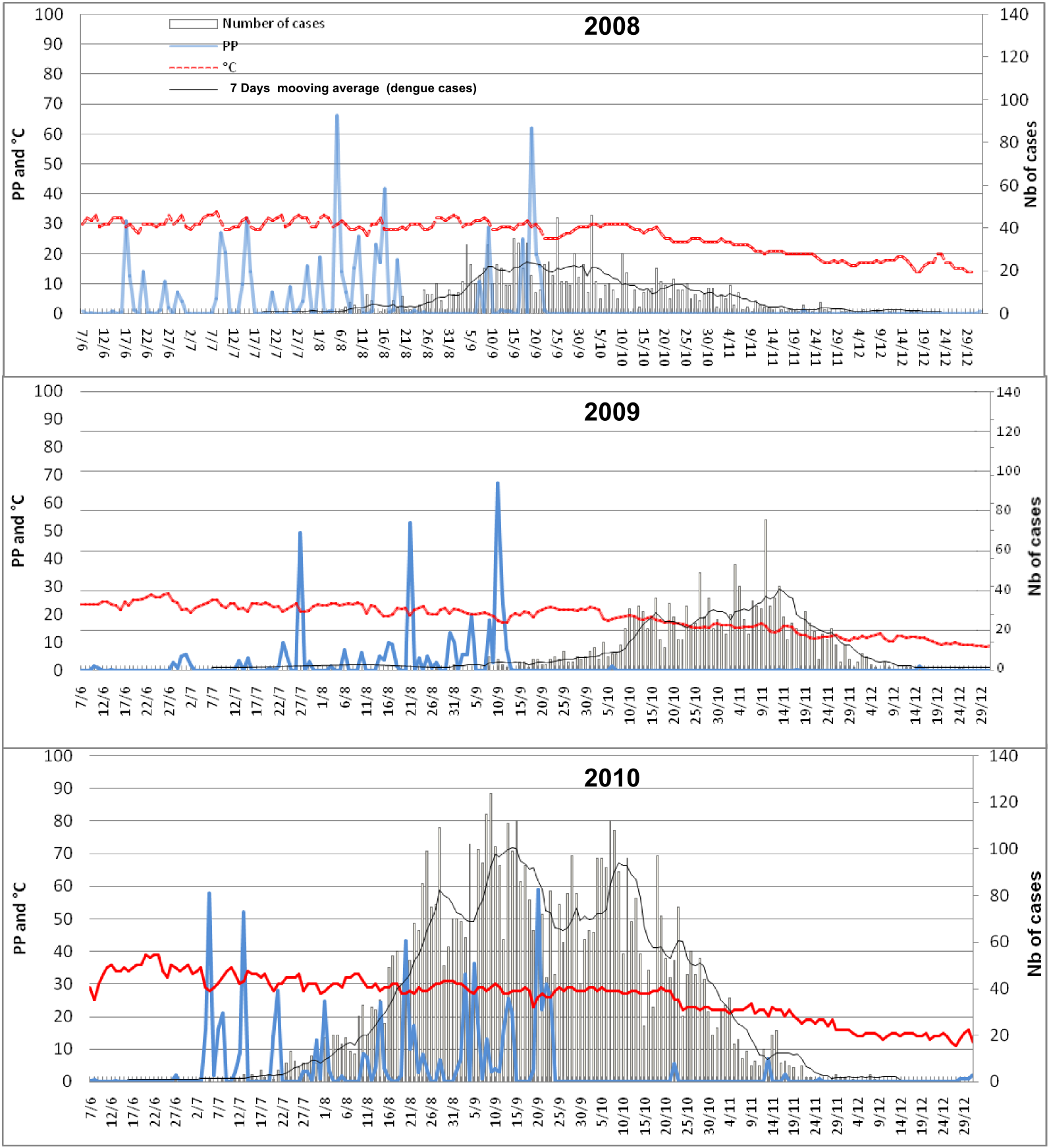

Supplement: S2 Fig — (TIF) [file pone.0146539.s002.tif]
