# Supplementary material for: The Spread of Dengue in an Endemic Urban Milieu–The Case of Delhi, India
Source: PLoS One. 2016 Jan 25;11(1):e0146539. doi: 10.1371/journal.pone.0146539 (PMC4726601; doi:10.1371/journal.pone.0146539)
Supplement: S3 Fig — (DOCX) [file pone.0146539.s003.docx]

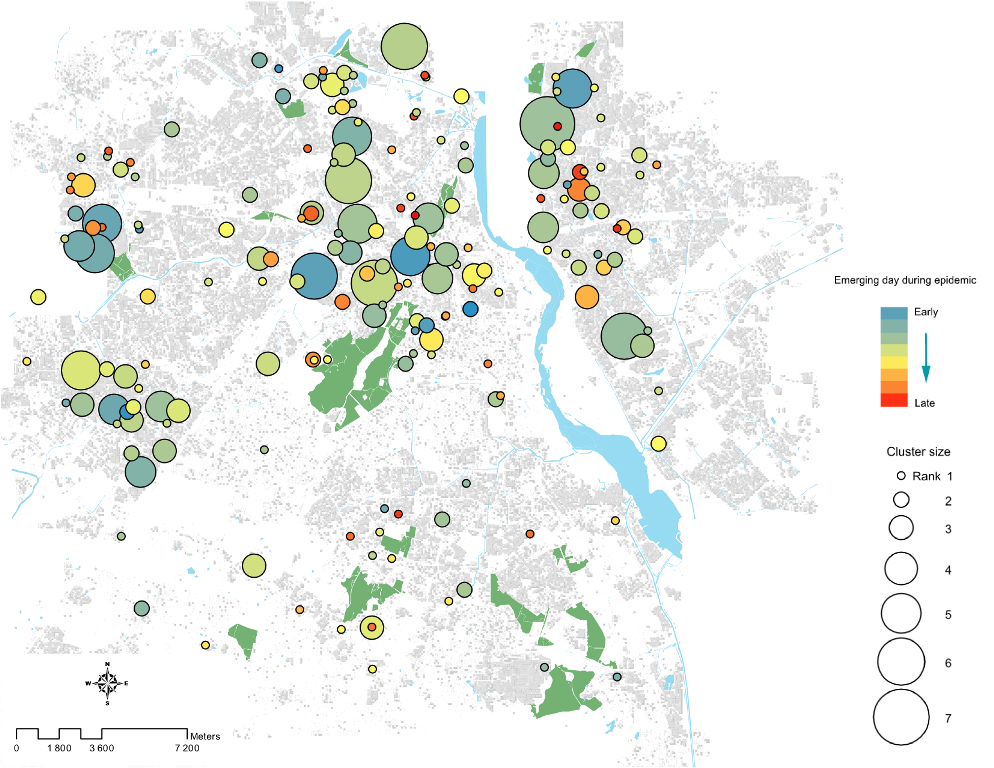

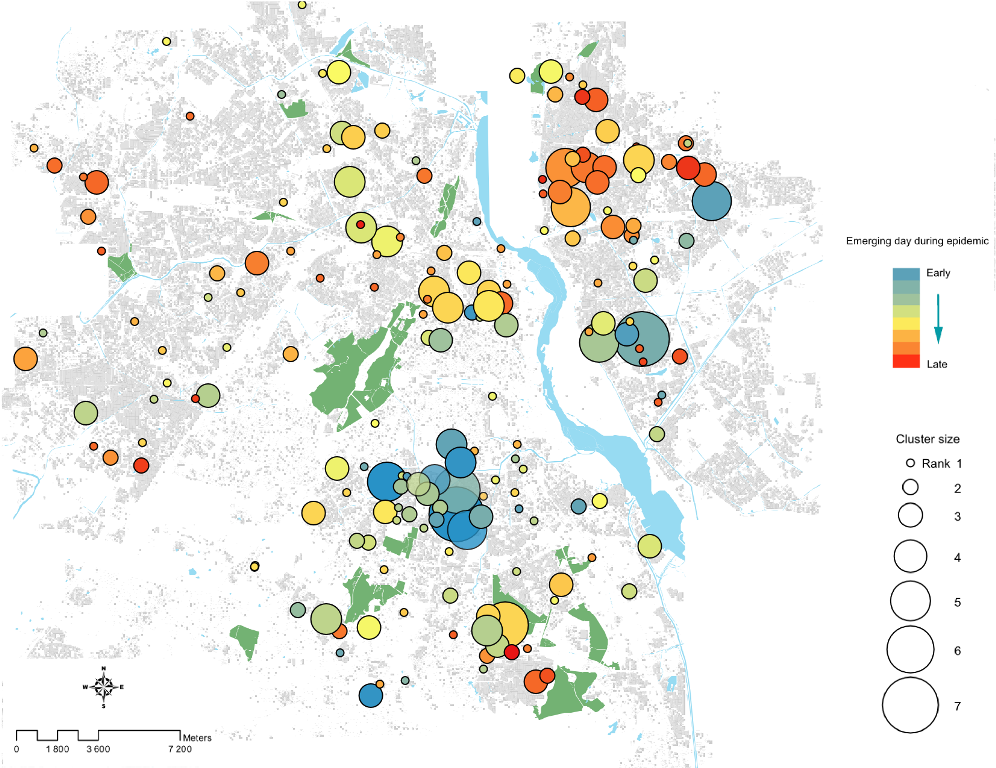

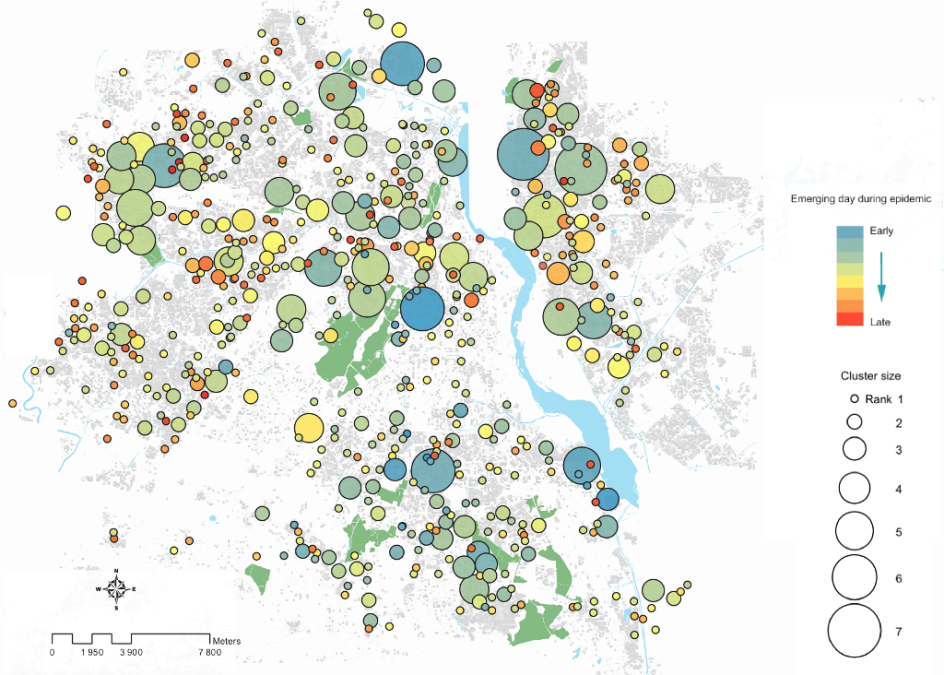


Sup. Material 4: Spatio temporal clusters detected in Delhi in 2008, 2009 and 2010: size and emerging day for each cluster.
