# Supplementary material for: The Spread of Dengue in an Endemic Urban Milieu–The Case of Delhi, India
Source: PLoS One. 2016 Jan 25;11(1):e0146539. doi: 10.1371/journal.pone.0146539 (PMC4726601; doi:10.1371/journal.pone.0146539)
Supplement: S1 File — (DOC) [file pone.0146539.s004.doc]

Figures showing the Gaussian fit of the number of cases per day per year (A-C) and the number of cases per cluster per day per year (D-F).


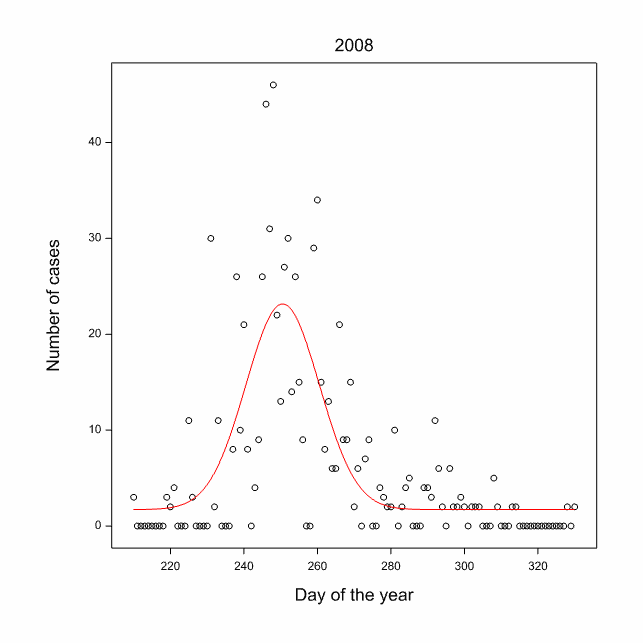

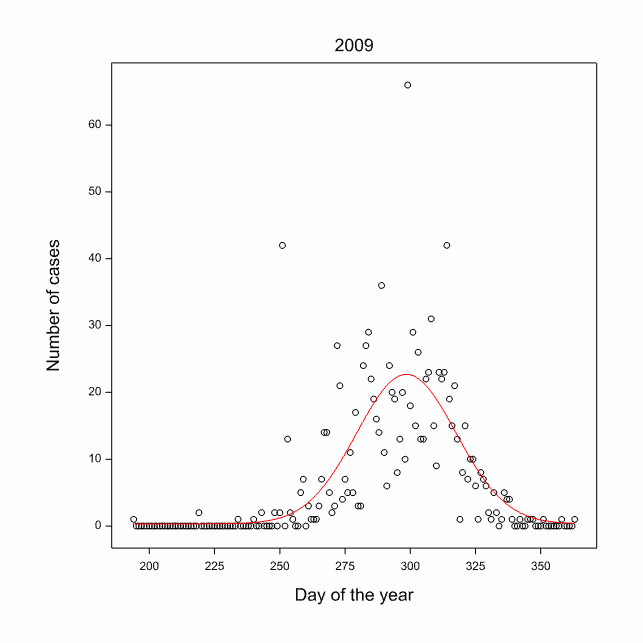

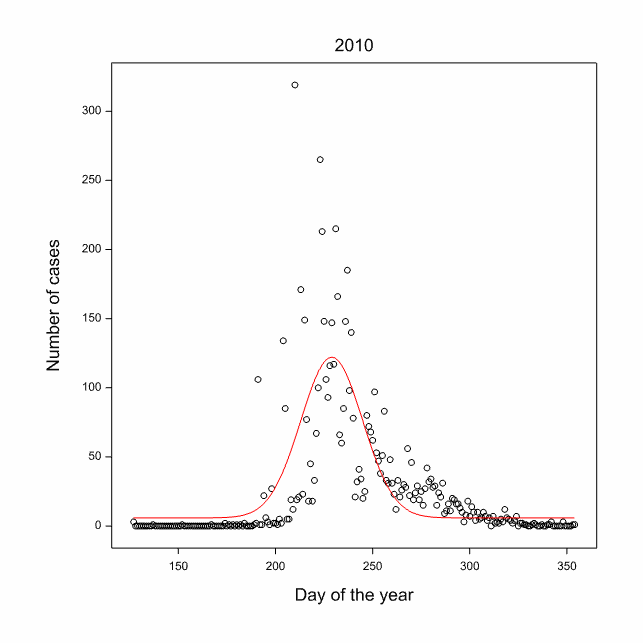

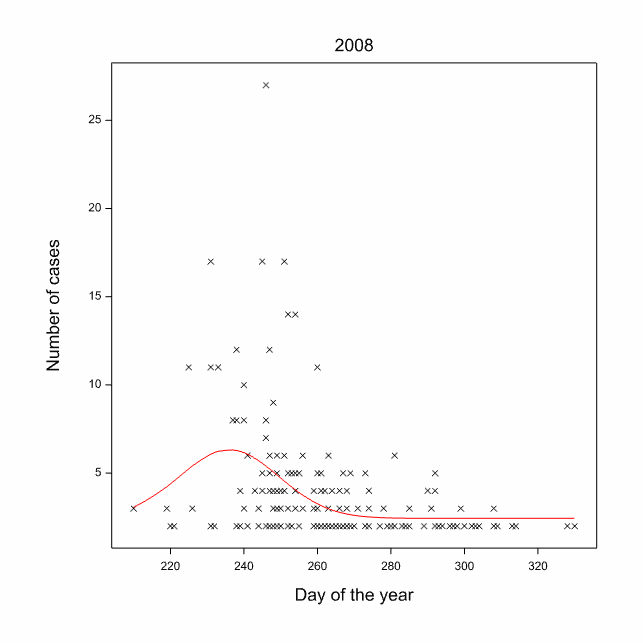

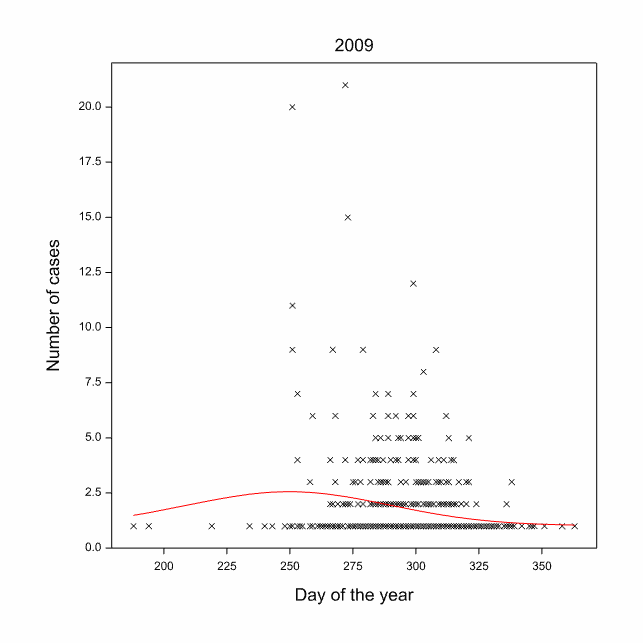

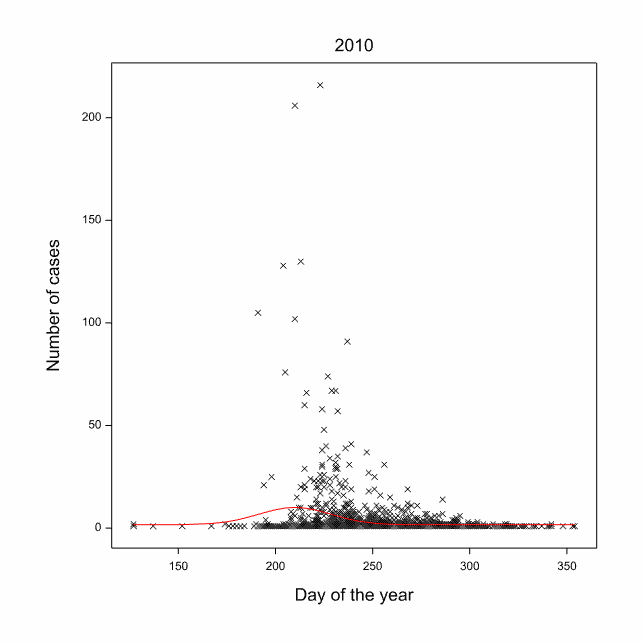


A

B

C

D

E

F
