# Supplementary material for: Correction: Correction: The Spread of Dengue in an Endemic Urban Milieu-The Case of Delhi, India
Source: PLoS One. 2016 Jul 5;11(7):e0158931. doi: 10.1371/journal.pone.0158931 (PMC4933359; doi:10.1371/journal.pone.0158931)
Supplement: S1 File — (PDF) [file pone.0158931.s001.PDF]

CORRECTION

Correction: The Spread of Dengue in an Endemic Urban Milieu-The Case of Delhi, India

The *PLOS ONE* Staff

The authors are listed out of order. The publisher apologizes for the error. Please view the correct author order, affiliations, and citation here:

Olivier Telle[1,2,4], Alain Vague[3], N.K Yadav[6], B. Lefebvre[3], A. Cebeillac[3,4], B.N. Nagpal[5], Eric Daudé[3,4], Richard E. Paul[1,2]

1 Centre National de la Recherche Scientifique, Unité de Recherche Associée 8204 Géographie-cités, Paris, France, 2 Institut Pasteur, Functional Genetics of Infectious Diseases Unit, Department of Genomes and Genetics, Paris, France, 3 Centre National de la Recherche Scientifique, Unité Mixte de la de Recherche 6266, IDEES, Rouen, France, 4 Centre de Sciences Humaines, Delhi, India, 5 National Institute of Malaria Research, Delhi, India, 6 Municipal Corporation of Delhi, Delhi, India

Telle O, Vaguet A, Yadav NK, Lefebvre B, Cebeillac A, Nagpal BN, et al. (2016) The Spread of Dengue in an Endemic Urban Milieu–The Case of Delhi, India. PLoS ONE 11(1): e0146539. doi:10.1371/journal.pone.0146539

In the Author Contributions section, Richard E. Paul (RP) should be listed as one of the persons who contributed reagents/materials/analysis tools.

Reference

1. Telle O, Vaguet A, Yadav NK, Lefebvre B, Daudé E, Paul RE, et al. (2016) The Spread of Dengue in an Endemic Urban Milieu–The Case of Delhi, India. PLoS ONE 11(1): e0146539. doi:10.1371/journal.pone.0146539 PMID: 26808518

CrossMark
click for updates

OPEN ACCESS

Citation: The *PLOS ONE* Staff (2016) Correction: The Spread of Dengue in an Endemic Urban Milieu-The Case of Delhi, India. PLoS ONE 11(3): e0152847. doi:10.1371/journal.pone.0152847

Published: March 29, 2016

Copyright: © 2016 The PLOS ONE Staff. This is an open access article distributed under the terms of the Creative Commons Attribution License, which permits unrestricted use, distribution, and reproduction in any medium, provided the original author and source are credited.
